# Robot-Guided Microballoon Compression of the Trigeminal Nerve for the Treatment of Trigeminal Neuralgia Caused by Fibrous Dysplasia of the Skull: A Case Report and Literature Review

**DOI:** 10.7759/cureus.74376

**Published:** 2024-11-24

**Authors:** Xingyuan Ma, Yicheng Luo, Tingzhen Deng, Maohua Zheng

**Affiliations:** 1 Neurosurgery, The First School of Clinical Medicine, Lanzhou University, Lanzhou, CHN; 2 Neurosurgery, The First Hospital of Lanzhou University, Lanzhou, CHN

**Keywords:** foramenovale, microballoon compression, robot-guided, skull fibrous dysplasia syndrome, trigeminal neuralgia

## Abstract

Cranial fibrous dysplasia (FD) syndrome is a benign, rare, and idiopathic skeletal disorder characterized by the replacement and expansion of medullary bone by disorganized fibro-osseous tissue. Trigeminal neuralgia (TN) is most commonly caused by vascular compression at the trigeminal nerve root entry zone. Secondary TN caused by cranial FD syndrome is extremely rare. This article reports a case of cranial FD syndrome in which the lesion resulted in posterior cranial fossa narrowing, leading to the occurrence of TN. We located the patient's foramen ovale with robotic navigation, and we effectively treated the patient's TN with balloon compression.

## Introduction

Craniofacial fibrous dysplasia (CFD) is a non-hereditary benign bone lesion, accounting for 7% of all bone lesions, with 70% affecting a single cranial bone and 30% involving multiple cranial bones [1]. CFD is more common in children and adolescents and occurs more frequently in females than males. It is generally considered self-limiting, active before puberty, and ceasing growth in adulthood. The cause remains unknown [1,2]. Pathologically, normal bone is replaced by immature bone tissue and fibrous connective tissue. Clinically, CFD is classified into three types: cranial vault, cranial base, and combined type [1].

Trigeminal neuralgia (TN) is characterized by paroxysmal, shock-like pain in one or more branches of the trigeminal nerve, often triggered by touch. Some patients also experience continuous pain. TN is divided into primary (PTN) and secondary (STN) forms [3]. The primary mechanism involves demyelination of the primary sensory afferents of the trigeminal nerve at the root entry zone, often caused by neurovascular conflict, leading to morphological changes like nerve root compression. However, not all PTN patients exhibit such changes. STN is caused by multiple sclerosis or space-occupying lesions affecting the trigeminal nerve [1].

CFD rarely leads to TN [4]. Previous case studies suggest morphological changes in the posterior cranial fossa leading to vascular compression of the trigeminal nerve root [4]. Hearing loss in the patient was considered related to temporal bone fibrous dysplasia (FD), although CT internal auditory canal, temporal bone plain + 3D reconstruction scans showed no significant abnormalities [5]. A study by Boyce et al. on the correlation between hearing loss and otologic outcomes in FD suggests that hearing loss is common in craniofacial FD, with most cases being mild to moderate. It is typically caused by FD compressing the ossicular chain and the elongation of the internal auditory canal (IAC), while narrowing of the external auditory canal (EAC) and invasion of the ear capsule are less common [6]. In order to further clarify the diagnosis and determine the etiology of TN and the surgical treatment plan, a head MRI scan was performed. The size and morphology of the skull were normal, with diffuse thickening of the skull bones, distortion of the trigeminal nerve course bilaterally; unclear relationship with blood vessels; and distortion of the course of the facial nerve, auditory nerve, glossopharyngeal nerve, abducens nerve, and buccal nerve. Based on the MRI manifestations, the imaging department considered this patient to have abnormal bone fiber proliferation disorder with skull base depression.

## Case presentation

A 55-year-old male presented with intermittent left mandibular pain without an obvious cause 10 years ago, which worsened in the past two months, accompanied by left neck pain and tongue tip pain. The pain, described as knife-like, lasted from a few minutes to several hours and occurred several times a day. Touching the painful area, speaking, or chewing did not affect the pain. There were no associated symptoms such as dizziness, headache, nausea, vomiting, altered consciousness, blurred vision, chills, or fever. The patient had been taking ibuprofen for six months without relief. Seeking further treatment, he was admitted to our hospital with a diagnosis of TN.

**Figure 1:**
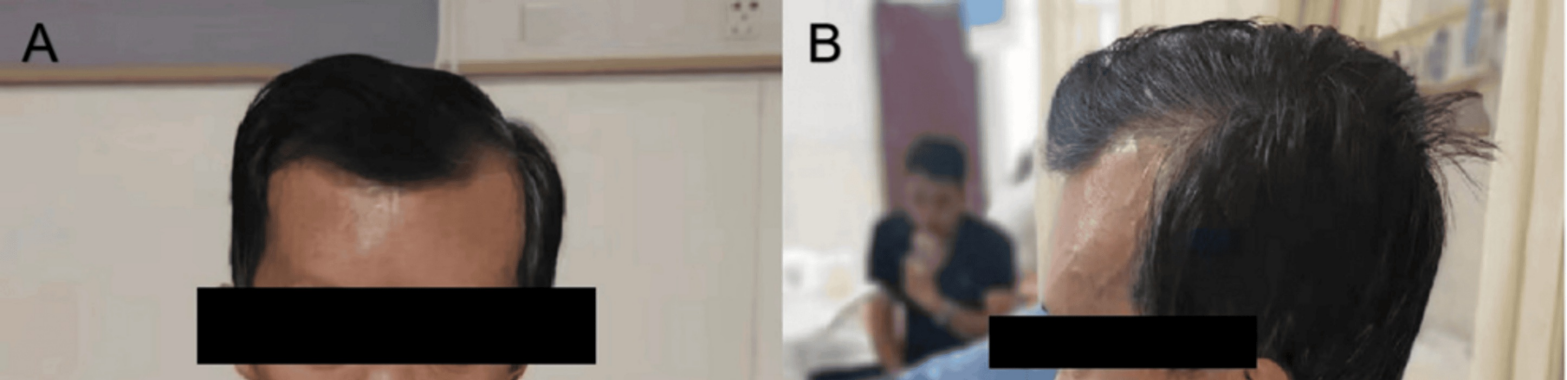
Preoperative clinical of patients with trigeminal nerve for the treatment of trigeminal neuralgia caused by fibrous dysplasia (A) Preoperative anterior view. (B) Preoperative lateral views.

On examination, a cystic swelling of the head was noted (Figure 1). The patient, with no significant symptoms and a slight decrease in strength, was admitted to the neurosurgery department. He had no history of trauma, surgery, or notable family history. Laboratory tests were normal (Table 1).

**Table 1:** Blood routine (venous blood) test and biochemical results of the case The laboratory tests of the patient showed that both the blood routine (venous blood) test results and the biochemical results were normal. In the table of the blood routine (venous blood) test results, WBC: white blood cell; RBC: red blood cell; Hb: hemoglobin; Hct: hematocrit; MCHC: mean corpuscular hemoglobin concentration; Plt: platelet; LY%: lymphocyte percentage; NE%: neutrophil granulocyte percentage; LYMPH#: lymphocyte absolute value; PCT: plateletcrit. In the table of the biochemical results, AST: aspartate aminotransferase; ALT: alanine aminotransferase; TP: total protein; ALB: albumin; ALP: alkaline phosphatase; GGT: γ-gamma glutamyltransferase; Urea: ureophil; Crea: creatinine; K: kalium; Na: sodium; CL: chlorine; Ca: calcium; TC: total cholesterol; TG: triglyceride; HDL-C: high-density lipoprotein; LDL-C: low-density lipoprotein; apoB: apolipoprotein B; CK: creatine kinase; HCY: homocysteine; CO_2_: carbon dioxide.

Blood routine (venous blood) test results of the case			
Variables	Preoperative result	Unit	Reference interval
WBC	3.06	10^9^/L	3.5-9.5
RBC	4.75	10^12^/L	4.3-5.8
Hb	135	g/L	130-175
Hct	44.3	%	40-50
MCHC	305	g/L	316-354
Plt	165	10^9^/L	125-350
LY%	14	%	20-50
NE%	76.8	%	40-75
LYMPH#	0.43	10^9^/L	1.1-3.2
PCT	0.166	%	0.169-0.374
Biochemical results of the case			
Variables	Preoperative result	Unit	Reference Interval
AST	31	U/L	15-40
ALT	12	U/L	9-50
TP	67.1	g/L	65-85
ALB	44.6	g/L	40-55
ALP	5082.6	U/L	45-125
GGT	19.8	U/L	10-60
Urea	3.56	mmol/L	3.1-8.0
Crea	56	μmol/L	57-97
K	3.99	mmol/L	3.5-5.5
NA	139.1	mmol/L	137-147
CL	103.6	mmol/L	99-110
Ca	2.17	mmol/L	2.11-2.52
TC	5.43	mmol/L	3.6-5.7
TG	2.7	mmol/L	0.8-1.8
HDL-C	1.35	mmol/L	0.8-1.8
LDL-C	3.5	mmol/L	1.55-3.7
apoB	1.15	g/L	0.8-1.1
CK	76	U/L	50-310
HCY	19.1	μmol/L	0-15
CO2	30.2	mmol/L	20-29

**Figure 2:**
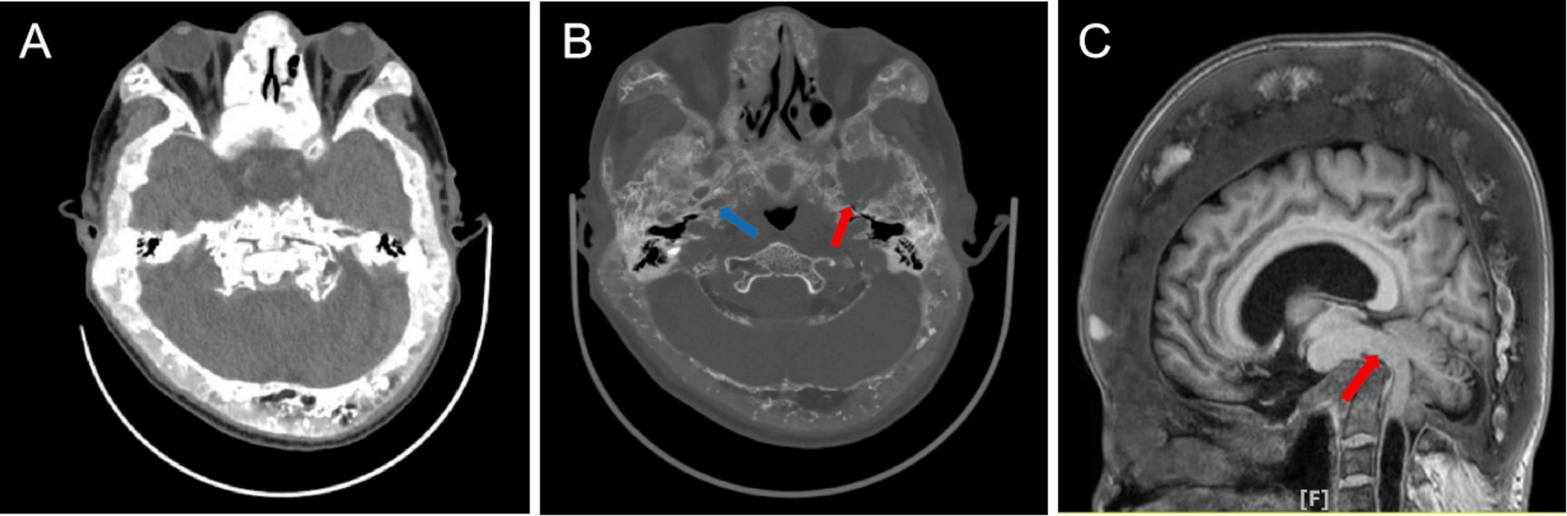
Imaging manifestations (A, B) Preoperative CT of the head. (A) Diffuse thickening of the cranial bones and uneven bone density was seen on 1.25 mm Stnd. (B) The affected foramen ovale (red arrow) appeared less distinct compared to the opposite side(blue arrow) was seen on the 1.25 mm bone. (C) MRI scans showed diffuse thickening of the cranial bones, primarily in the outer plate, with nodular and cord-like mixed signals within the diploic space. Other findings included clear gray-white matter differentiation, slightly elongated T1 and T2 signal bands around the lateral ventricles, normal ventricular and cisternal widening, reduced cerebellar volume, and brainstem distortion (red arrow). The cerebellum extends upward through the tentorial notch and downward through the foramen magnum, reaching the mid-level of the second cervical vertebra, suggesting a potential Chiari II malformation. In addition, there is evidence of syringomyelia or a cerebrospinal fluid cavity starting below C2.

A head CT scan (including plain and enhanced scans) showed diffuse thickening of the cranial bones and uneven bone density (Figure 2A). Bilateral internal auditory canals, temporal bone sweeping + three-dimensional reconstruction were performed: bilateral external auditory canals were patent; the size and shape of the middle ear cavity did not show any obvious abnormality; the middle ear cavity did not see abnormal early density filling, the auditory ossicular chain was intact, and the hammerstone and anvil articular space existed; the shape of the bony cochlea and the semicircular canals was as normal; the internal auditory canals did not show any obvious dilation or narrowing. Bilateral temporal bone mastoid pneumatization was fair, and the atrial space was clear.

CT data suggested FD, but a definitive diagnosis was not possible. Reviewing the literature, we hypothesized that the facial pain could be explained by compression of the trigeminal nerve root at the cranial base. The affected foramen ovale appeared less distinct compared to the opposite side (Figure 2B). However, it is crucial to distinguish it from primary TN, which has a low chance of being combined with cranial FD syndrome.

An MRI of the head showed that the size and shape of the skull were normal and diffuse thickening of the cranial bones, primarily in the outer plate, with nodular and cord-like mixed signals within the diploic space. Other findings included symmetric cerebral. The distance from the target to the trigeminal crossing point was measured on the robotic navigation interface, indicating the required catheter insertion depth. The second breakthrough sensation indicated entry into Meckel's cave. After confirmation with fluoroscopy, 0.3 ml of contrast medium was slowly injected, and the balloon shape was observed. The balloon was then filled to 0.7 ml and compressed for one minute. The balloon catheter and puncture needle were then removed, and the area was covered with a sterile dressing. The patient experienced no adverse reactions during surgery and recovered consciousness post-operatively, returning to the ward. Pain was absent post-surgery, and the patient was discharged three days later. Eight weeks post-surgery, telephone follow-up reported no special discomfort. Hemispheres, clear gray-white matter differentiation, slightly elongated T1 and T2 signal bands around the lateral ventricles, normal ventricular and cisternal widening, centered midline structures, reduced cerebellar volume, and brainstem distortion were observed (Figure 2C). There were no abnormal signals in the brain parenchyma, and the pituitary gland appeared normal. The sagittal view showed a linear fluid area within the cervical cord, suggesting spinal cord compression and thinning. The bilateral trigeminal nerves were distorted and the relationship with surrounding vessels was unclear. The diagnosis was CFD combined with TN. The rest of the laboratory tests were normal.

Surgical details

**Figure 3:**
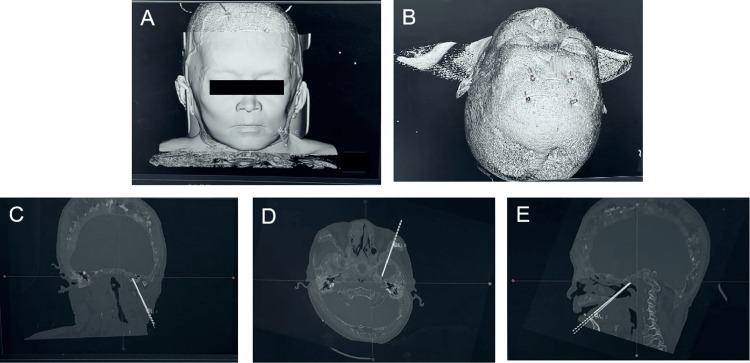
Intraoperative clinical images Registration was performed using four bony landmarks with the robotic pointer. The surgical plan, based on fused MRI and CT images, determined the needle path angles in the coronal (C), horizontal (D), and sagittal planes (E).

**Figure 4:**
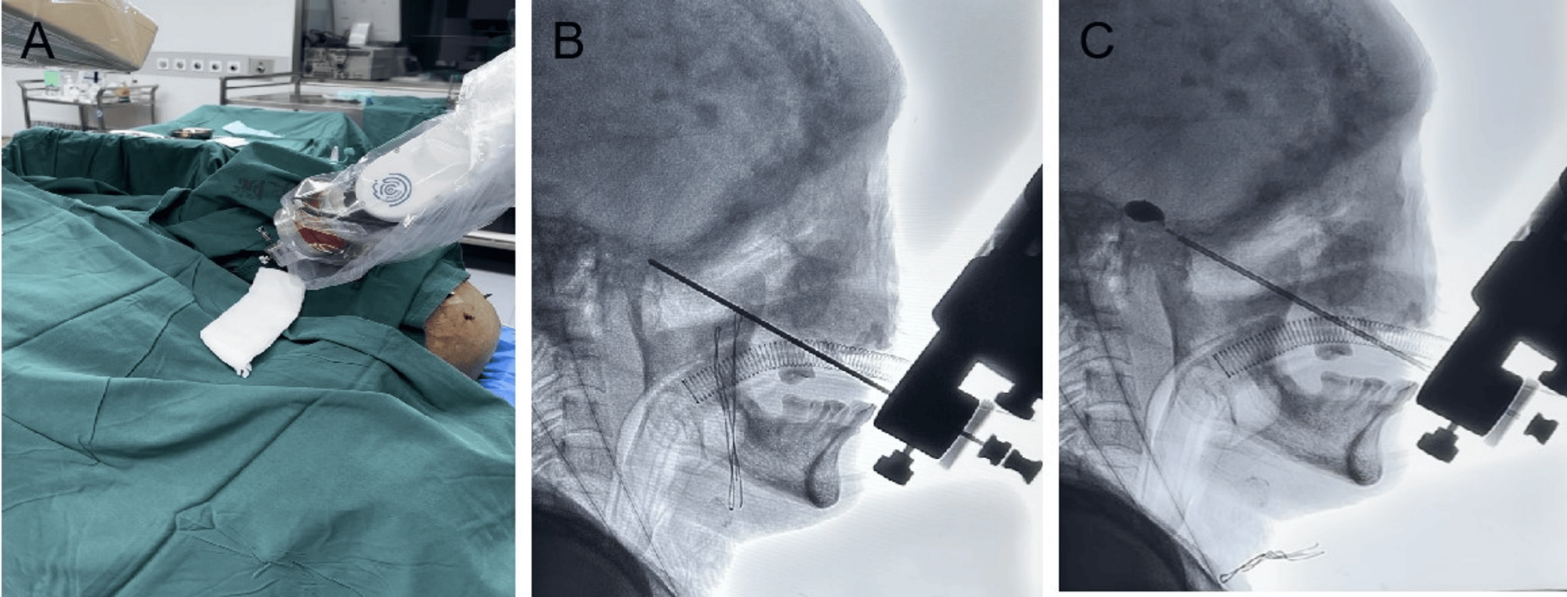
Intraoperative clinical images (A) Robot-guided location of foramen oval. During the operation, the position and shape of the puncture needle (B) and the balloon (C) after expansion were verified successively. The balloon was in a typical "pear-shaped" shape.

The patient's head was fixed using a three-pin frame and connected to the Huada neurosurgical robot's linkage arm. Registration was performed using four bony landmarks, with the robotic pointer marking the points (Figure 3A, 3B). Based on the fused MRI and CT images, the spatial positions of the head landmarks were acquired through the photonic encoder of the built-in position sensor in the robotic arm, automatically matching the patient's cranial position with the preoperative imaging data. The surgical plan was formulated, determining the needle path angles in the coronal, horizontal, and sagittal planes (Figure 3C, 3D, 3E). The optimal puncture path from the inner edge of the mandibular angle through the foramen ovale to Meckel's cave was established, with an average error of 0.91 mm. The patient was positioned supine on the angiography table, under general anesthesia with a laryngeal mask. After verifying the registration points, routine disinfection was performed, and the robotic arm's pointer was replaced with a trigeminal nerve puncture sleeve, positioning it for the puncture procedure. The robotic arm’s axial position and planned trajectory perfectly aligned. A 20 ml syringe needle was used to puncture the skin. The puncture point was located 2.5 cm lateral and 1.5 cm superior to the left oral commissure, directed toward a point 2 cm anterior to the bilateral external auditory canal line and the inner edge of the left pupil (Figure 4A). Adjustments were made under C-arm fluoroscopy until the foramen ovale was reached, indicated by a distinct breakthrough sensation (Figure 4B). The robot enabled precise localization of the foramen ovale in this patient with abnormal cranial anatomy. During PBC surgery, significant hemodynamic fluctuations are often observed, leading to severe bradycardia, even cardiac arrest, and sudden increases in blood pressure, known as trigeminal-cardiac reflex (TCR). Prophylactic administration of atropine is typically recommended to prevent or reduce TCR-related bradycardia, while sodium nitroprusside is used to prevent TCR-associated hypertension. In addition, a combination of sodium nitroprusside and atropine is also employed for TCR pre-treatment. This triggered a trigeminal reflex (elevated blood pressure and decreased heart rate), which was managed with intravenous antihypertensive medication (target systolic pressure < 140 mm Hg) and atropine (target heart rate > 60 bpm). Once the target was reached, the inner core was removed, and a balloon catheter was placed.

The distance from the target to the trigeminal crossing point was measured on the robotic navigation interface, indicating the required catheter insertion depth. The second breakthrough sensation indicated entry into Meckel's cave. After confirmation with fluoroscopy, 0.3 ml of contrast medium was slowly injected, and the balloon shape was observed (Figure 4C). The balloon was then filled to 0.7 ml and compressed for one minute. The balloon catheter and puncture needle were then removed, and the area was covered with a sterile dressing. The patient experienced no adverse reactions during surgery and recovered consciousness post-operatively, returning to the ward. Pain was absent post-surgery, and the patient was discharged three days later. Eight weeks post-surgery, telephone follow-up reported no special discomfort.

## Discussion

CFD is a slowly progressing benign lesion affecting multiple bones [7]. It presents as FD when normal bone development halts at the membranous ossification stage, leading to the proliferation of fibrous connective tissue in cranial bones. Patients often seek medical attention due to local cranial protrusions or cranial nerve damage [2,8]. Among the cranial nerve damages the optic nerve has the highest incidence [9]. The patient had been ill for more than 30 years and had not been treated with surgery or other related treatments.

TN incidence varies, with 4.3 to 27 new cases per 100,000 people annually. Women are more frequently affected, and the incidence increases with age. Lifetime prevalence has been estimated at 0.16-0.3% in population-based studies. The mean age of onset is 53 years for classic TN and 43 years for secondary TN, but the age of onset can vary from early to old age. In studies based on tertiary care, STN accounted for 14-20% of patients with TN [10].

Neurovascular conflict at the trigeminal root entry zone is a potential cause, although not all patients exhibit this. However, the specific etiology of NVC is unknown. Furthermore, it has been shown that NVC has been demonstrated in healthy subjects and that TN can occur in patients without vascular compression, suggesting that NVC alone is not sufficient to produce TN. suggesting that alterations in the morphology and volume of the posterior cranial fossa may play a role in the development of NVC [4]. Several cases of TN have been associated with Paget's disease, rocky bone distortion, Chiari malformation, chondrodysplasia, and Dandy-Walker malformation, including disorders in which the posterior cranial fossa is crowded due to lesions or malformations, such as the combined cranial fibroplastic syndromes of this patient [4,11].

Bollen et al. have reported a case of an elderly woman with anomalous bone fibrosis of the base of the skull in combination with TN [11]. In addition, it is well known that there is a female predominance in TN possibly due to the fact that the posterior cranial fossa is more crowded in women than in men [4]. TN secondary to CFD is a specific type of neuralgia with different clinical features from other types of TN [12]. TN secondary to CFD usually presents as severe paroxysmal pain in the face, which may spread along the area of distribution of the trigeminal nerve, affecting the forehead, around the eyes, nose, lips, and chin [12]. The pain may appear suddenly, last anywhere from a few seconds to a few minutes, and then suddenly disappear [12,13]. This pain may be triggered by daily activities such as touching, hot or cold stimuli, eating, or talking, causing great distress to the patient's daily life. In addition to pain, patients with TN secondary to CFD may also experience facial numbness, abnormal sensation, and muscle weakness or atrophy [12]. These symptoms may be related to changes in bone structure and nerve compression caused by abnormal proliferation of cranial fibers [12,13]. TN secondary to CFD has some unique clinical features compared to other types of TN. First, this pain is usually associated with abnormal proliferation of cranial and facial bones, whereas other types of TN may be associated with a variety of factors such as age, vascular compression, and inflammation [3]. Second, TN secondary to CFD may present in childhood or adolescence, while other types of TN are more common in adults. In addition, patients with TN secondary to CFD usually have abnormal enlargement of the skull and facial bones detected on imaging, whereas other types of TN do not always show this [12,13].

CT examination for the diagnosis of this disease to X-ray film has obvious superiority because CT can not only carry out three axial positions of the bone window scanning but also can carry out three-dimensional reconstruction and high resolution, can accurately determine the scope of the lesion and its relationship with the surrounding structures, can clearly show the lesion area of the trigeminal nerve that is involved and the degree of compression, and can guide the selection of surgical program. The MRI signal of poor bone fiber structure is variable, but in this disease, MRI can show the distortion of the trigeminal nerve, and the relationship with blood vessels is not clear; the facial nerve, auditory nerve, glossopharyngeal nerve, abducens nerve, and buccal nerve are distorted. The type of TN is clarified and the subsequent surgical plan is determined. Relative to this patient, i.e., patients with cranial FD syndrome due to the reduced morphology and the volume of the posterior cranial fossa, studies have also confirmed that the small volume of the posterior cranial fossa is a risk factor for neurovascular conflict [4]. This patient's TN symptoms were caused by vascular compression at the REZ and were influenced by anatomical factors caused by cranial FD syndrome, such as the small volume of the posterior cranial fossa.

The treatment of TN is categorized into medical treatment and surgical treatment. Oral medication is the preferred treatment for TN, and carbamazepine and gabapentin are more commonly used. Surgery can be chosen for patients who cannot tolerate the side effects of oral medication or those who cannot tolerate the side effects of medication [10]. Currently, there are three mainstream surgical procedures for the treatment of TN: cranial microvascular decompression, radiofrequency ablation of the trigeminal semilunar ganglion, and balloon compression [7]. However, so far, none of these treatments has been able to combine safety, efficacy, and low recurrence rate at the same time. Open microvascular decompression (MVD) is still one of the most classic surgical methods for the treatment of TN, but due to its long surgical time, high trauma, and high surgical cost, it is difficult to be accepted by most patients, and there are still a variety of postoperative complications [14]. Craniotomy microvascular decompression surgery is traumatic and has a long postoperative recovery period; patients with this disease are also unsuitable for craniotomy due to diffuse thickening of the bones of the skull and uneven bone density. Radiofrequency thermocoagulation (RFT) of the trigeminal nerve requires the patient to operate under local anesthesia, with severe intraoperative pain and a high recurrence rate and is usually indicated for patients older than 70 years of age who have already undergone ineffective microvascular decompression or who have pain recurrence [15]. Percutaneous balloon compression can be performed under general anesthesia without discomfort during the operation, and the postoperative recovery is faster than that of open microvascular decompression, so it has been favored by more and more doctors and patients [14,15]. Traditional manual puncture mainly relies on C-arm X-ray lateral images, which cannot directly visualize the foramen ovale or Meckel's cavity. The foramen ovale is typically located using the apex of the petrous bone and tactile sensation during puncture but with less precision.

Liu et al. also pointed out that although rare, severe complications from PMC include carotid-cavernous fistulas and other vascular issues [8]. By contrast, robotic assistance can help precisely locate the foramen ovale on CT and Meckel's cavity on MRI, guiding the balloon catheter placement depth. This method enables accurate puncturing and catheter placement, achieving individualized procedures. The rapid development of neurosurgical robots in recent years provides satisfactory and reproducible solutions for this purpose [16]. In this patient, due to cranial fibrous anomalous hyperplasia, the location of the foramen ovale could not be routinely and accurately localized, and the depth of puncture was not well defined. The fusion of CT and MRI images significantly enhances the precision of localizing the foramen ovale by combining complementary imaging data. CT provides clear anatomical details of bony structures, such as the foramen ovale, while MRI offers detailed visualization of soft tissues, including Meckel's cave. This integration allows accurate identification of the target region and precise measurement of the distance from the foramen ovale to the distal end of Meckel's capsule. Such precision minimizes procedural risks and optimizes the placement of the balloon catheter during surgery. Precise puncture and placement of the catheter can be achieved with individualized operation. According to the CT axial and coronal cerebral sickle adjusted images for the standard axial and coronal images, the three-dimensional images were used to find the foramen ovale on the two-dimensional images. A retrospective study by Brown et al. showed that in 56 cases with a balloon pressure of 750-1250 mm Hg and a compression duration of 1.5 minutes, 92% of the patients had immediate postoperative pain relief [17]. There is a lack of clarity on how to quantify and individualize balloon pressure. Studies have shown that with intra-balloon injection of 1 ml of fluid compression for 1 min, postoperative pain was relieved, but all of them showed facial numbness on the operated side, half of the operated side of the tongue deep and shallow sensory deficits, with the average follow-up of 11 months. With the repair of the nerves, only two of the 20 cases are still residual slight numbness of the face, and the remaining 18 cases of postoperative complications have disappeared [17].

With the continuous progress and innovation of medical technology, robotic navigation systems are increasingly used in the field of surgery. Its precise positioning and efficient operation performance have revolutionized surgery. Especially in complex craniofacial surgeries, the robotic navigation system, with its unique advantages, provides the surgeon with a more precise and safer surgical solution [17]. TN, as a common cranial nerve disease, is characterized by severe and unbearable pain, which seriously affects the quality of life of patients. By contrast, cranial fibrous hyperplasia is a rare cranial disease, which, as mentioned earlier, has a great impact on the common surgical treatments for TN in the clinic, making surgery much more difficult. Therefore, the surgical treatment of patients with TN combined with cranial fibrodysplasia has been a difficult and hot research topic in the medical field.

In recent years, with the continuous development of robotic navigation technology, its application in craniofacial surgery has gradually increased. The robotic navigation system can help the surgeon find the lesion site more accurately through precise image localization and navigation, reducing errors and injuries during surgery. At the same time, the robotic navigation system can also provide real-time surgical feedback and monitoring to ensure the safety and effectiveness of surgery. Therefore, the application of robotic navigation systems in the surgical treatment of patients with TN combined with cranial fibrous anomalies proliferation has important clinical value and research significance.

The study aims to evaluate the therapeutic effectiveness and safety of PMC surgery for patients with TN and cranial fibrous anomalies using robotic navigation. It focuses on specific outcomes, such as pain reduction, minimizing intraoperative nerve injury, and improving surgical precision. By analyzing preoperative, intraoperative, and postoperative data, the research seeks to assess the impact of robotic navigation on surgical techniques and complication prevention. The goal is to enhance safety, efficacy, and patient quality of life through innovative surgical approaches. It is hoped that this study will provide new ideas and methods for the surgical treatment of patients with TN combined with cranial fibrous anomalies proliferation. At the same time, this study will also explore the influence of the robotic navigation system on the operating skills of the surgeon, as well as the prevention and treatment of complications that may occur during surgery. It is hoped that this study will provide new ideas and methods for the surgical treatment of patients with TN combined with cranial fibrous anomalies proliferation, improve the safety and effectiveness of surgery, and bring better treatment results and quality of life to patients.

Robot-guided percutaneous trigeminal nerve semilunar ganglion balloon compression also has some inherent drawbacks, such as the high acquisition cost of robotic equipment, the need for preoperative import of imaging data, and pathway planning, which increases the complexity of implementation. In summary, robot-guided percutaneous trigeminal semilunar ganglion balloon compression is characterized by high clinical operational precision, small intraoperative fluctuations in vital signs, and satisfactory postoperative outcomes. In the future, with the continuous progress of technology, we expect that the robotic-guided surgical system can be applied in more fields and further improve the accuracy and safety of surgery. At the same time, we also need to pay attention to the ethical and legal issues that robotic surgery may bring, such as the operating authority of the surgical robot and the attribution of responsibility.

## Conclusions

In cases of TN caused by reduced posterior cranial fossa volume and shape, as well as neurovascular conflicts affecting cranial nerves and related structures, percutaneous trigeminal semilunar balloon compression is the most suitable treatment option. This approach is particularly effective for patients with abnormal cranial fibrous structures, as it adapts well to unique anatomical conditions, offering a safer and more effective therapeutic solution tailored to the individual patient's needs. The two major difficulties of traditional percutaneous trigeminal semilunar balloon compression (one is the puncture into the foramen ovale and the other is the placement of the balloon catheter into the Meckel's capsule) were made more difficult by the robot's software that could accurately measure the distance between the foramen ovale and the distal end of the Meckel's capsule to guide the depth of balloon catheter placement. However, more cases and studies are needed to confirm and explore these potential associations and the mechanisms behind them.

With the continuous progress and innovation of medical technology, robotic navigation systems are increasingly widely used in the field of surgery. Its precise positioning and efficient operation performance have revolutionized surgery. Especially in complex craniofacial surgeries, the robotic navigation system, with its unique advantages, provides doctors with more precise and safer surgical solutions. The effect of the application of a robotic navigation system in surgery is evaluated by observing and analyzing the indicators of patients before, during, and after surgery. In the surgical treatment of TN combined with cranial fibrous anomalies, this case utilizes robot-assisted surgery to locate the foramen ovale on CT images and the Meckel's cavity on MRI. It also accurately measures the distance between the foramen ovale and the distal end of Meckel's cavity, guiding the depth of balloon catheter placement. This approach allows for precise puncturing and catheter placement, achieving individualized procedures. In addition to improving puncture accuracy, robotic guidance also stabilizes the puncture pathway. Overall, it enhances the safety and effectiveness of surgery, leading to better treatment outcomes and quality of life for patients.
